# Baseline pro-inflammatory gene expression in whole blood is related to adverse long-term outcomes after transcatheter aortic valve replacement: a case control study

**DOI:** 10.1186/s12872-021-02186-0

**Published:** 2021-08-02

**Authors:** Deena S. Goldwater, Mei Leng, Arun Karlamangla, Teresa Seeman, David Elashoff, Jonathan M. Wanagat, David B. Reuben, Brian R. Lindman, Steve Cole

**Affiliations:** 1 Division of Cardiology, Department of Medicine, University of California, Los Angeles, CA USA; 2 Division of Geriatrics, Department of Medicine, University of California, Los Angeles, CA USA; 3 Department of Biostatistics, University of California, Los Angeles, CA USA; 4 Veterans Administration Greater Los Angeles Healthcare System, Los Angeles, CA USA; 5 Structural Heart and Valve Center, Vanderbilt University Medical Center, Nashville, TN USA; 6 Semel Institute for Neuroscience and Human Behavior, University of California, Los Angeles, CA USA

**Keywords:** Transcatheter aortic valve replacement, Geriatrics, Inflammation, Gene expression, Quality of life

## Abstract

**Background:**

Age-associated inflammation and immune system dysfunction have been implicated as mechanisms that increase risk for adverse long-term procedural outcomes in older adults. The purpose of this study was to investigate relationships between baseline inflammatory and innate antiviral gene expression and outcomes after transcatheter aortic valve replacement (TAVR) in older adults with severe aortic stenosis.

**Methods:**

We performed a retrospective case–control study comparing pre-procedural pro-inflammatory and Type 1 interferon (IFN) gene expression in 48 controls with favorable outcomes (alive 1 year after TAVR with improved quality of life [QoL]) versus 48 individuals with unfavorable outcomes (dead by 1 year or alive at 1 year but with reduced QoL). Gene expression was evaluated in whole blood via (1) pre-defined composite scores of 19 inflammation-associated genes and 34 Type I IFN response genes, and (2) pro-inflammatory and antiviral transcription factor activity inferred from promotor based bioinformatics analyses of genes showing > 25% difference in average expression levels across groups. All analyses were adjusted for age, gender, body mass index, diabetes, immunosuppression, cardiovascular disease (CVD), and frailty.

**Results:**

Relative to controls, those with unfavorable outcomes demonstrated higher expression of the pro-inflammatory gene composite prior to TAVR (p < 0.01) and bioinformatic indicators of elevated Nuclear Factor kB (p < 0.001) and Activator Protein 1 (p < 0.001) transcription factor activity, but no significant differences in Type I IFN-related gene expression.

**Conclusions:**

These results demonstrate that a pro-inflammatory state prior to TAVR, independent of CVD severity and frailty status, is associated with worse long-term procedural outcomes.

## Background

Due to increasing average life expectancy, along with the high incidence of cardiovascular disease in aging, more than half of all cardiovascular procedures are performed in adults over 65 years of age [1]. However, despite overall favorable safety profiles, older adults are more susceptible to adverse long-term outcomes such as mortality and deteriorating QoL after a procedure [2]. Although the presence of age-related risk factors (e.g. multimorbidity and frailty [3–5]) predisposes individuals to these unfavorable health trajectories, even those without these age-associated conditions may do poorly despite technically successful interventions [6]. The biological mechanisms and systems interactions contributing to this vulnerability are poorly understood.

Transcatheter aortic valve replacement (TAVR), a minimally invasive alternative to surgical aortic valve replacement, exemplifies the benefits and challenges of performing cardiovascular interventions in older adults and provides a discrete, real-world procedural stress model with which to explore the spectrum of resilience and vulnerability. Despite an average patient age of over 80 years, numerous randomized trials show survival and QoL benefit after TAVR for severe symptomatic aortic stenosis (AS) [7, 8]. However, accumulating evidence suggests that clinical risk factors, such as geriatric syndromes, influence long-term outcomes in addition to the Society for Thoracic Surgeon (STS) risk score [9–11]. Yet, the addition of these syndromes into clinical risk models has not fully accounted for the spectrum of observed outcomes.

Biomarkers of inflammatory and immune system function may provide additional insight into vulnerability to adverse outcomes after TAVR. Several studies have shown that elevated baseline serum levels of inflammatory and immune biomarkers predict unfavorable long-term TAVR outcomes. Specifically, indicators of generalized inflammation, (i.e., albumin, interleukin [IL] 6, IL8, and C-reactive protein [CRP]) are associated with worse 1- and 2-year outcomes [12–15]. Similarly, immune system dysfunction as measured by neutrophil-to-lymphocyte ratio or low levels of T-helper cells predict adverse procedural outcomes. Moreover, Lindman et al*.* (2015), recently showed that elevation of multiple serum indicators simultaneously significantly increases risk for unfavorable outcomes [15], implying that patterns of pre-TAVR inflammatory and immune system activity may be more informative with respect to long-term outcomes as compared to individual biomarkers.

One way to analyze patterns of inflammatory and immune system activity is to explore transcriptional activity of inflammatory and immune system-related genes measured in whole blood. Although such pathways have not been directly assessed in the context of TAVR outcome prediction, it is plausible that altered immune regulatory signaling may contribute to such effects because several common molecular pathways of inflammatory and immune system activity have been associated with both aging and chronic disease, and could thus increase vulnerability to adverse procedural outcomes [16–18]. In addition to assessing aspects of cellular function that may not be represented in circulating plasma markers, transcriptome profiles can be analyzed using bioinformatics strategies to identify the specific cellular signaling pathways that mediate differences in inflammatory biology and might serve as targets for future therapeutic interventions. Utilizing genome-wide transcriptomic analysis of whole blood may, therefore, provide an improved understanding of the inflammatory and immune system in patients undergoing TAVR and the relationship between these potentially dysregulated systems, chronic age-related disease, and procedural vulnerability.

In this study, we explore whether innate inflammatory and Type 1 interferon (IFN) antiviral gene expression prior to TAVR is related to adverse long-term outcomes using two different approaches to assessing inflammatory and antiviral activity in whole blood. The first approach utilized *a-priori* defined composite scores quantifying average expression levels for 19 pro-inflammatory genes and 34 genes involved in Type I IFN activity. The second approach utilized an alternative technique for testing the same general hypotheses, using bioinformatics analyses of pre-specified pro-inflammatory and antiviral transcription factor activity in the empirical transcriptomic correlates of long-term TAVR outcomes.

## Methods

### Patient population

This study takes advantage of previously collected clinical and biological data from an observational prospective cohort study designed to develop prediction models for patient-centered clinical outcomes in older adults with symptomatic severe AS undergoing TAVR; patients undergoing valve-in-valve procedures were excluded. Participating recruitment sites were Washington University in St.Louis, Cleveland Clinic Foundation, Intermountain Medical Center, Vanderbilt University, Universtiy of Texas Southwestern, Massachusetts General Hospital, Stanford University Medical Center, University of Utah School of Medicine, North Florida/South Georgia Veterans Health System, Morristown Medical Center, and Dartmouth Medical Center. All sites followed an IRB approved protocol.

### Sample selection

**Fig. 1:**
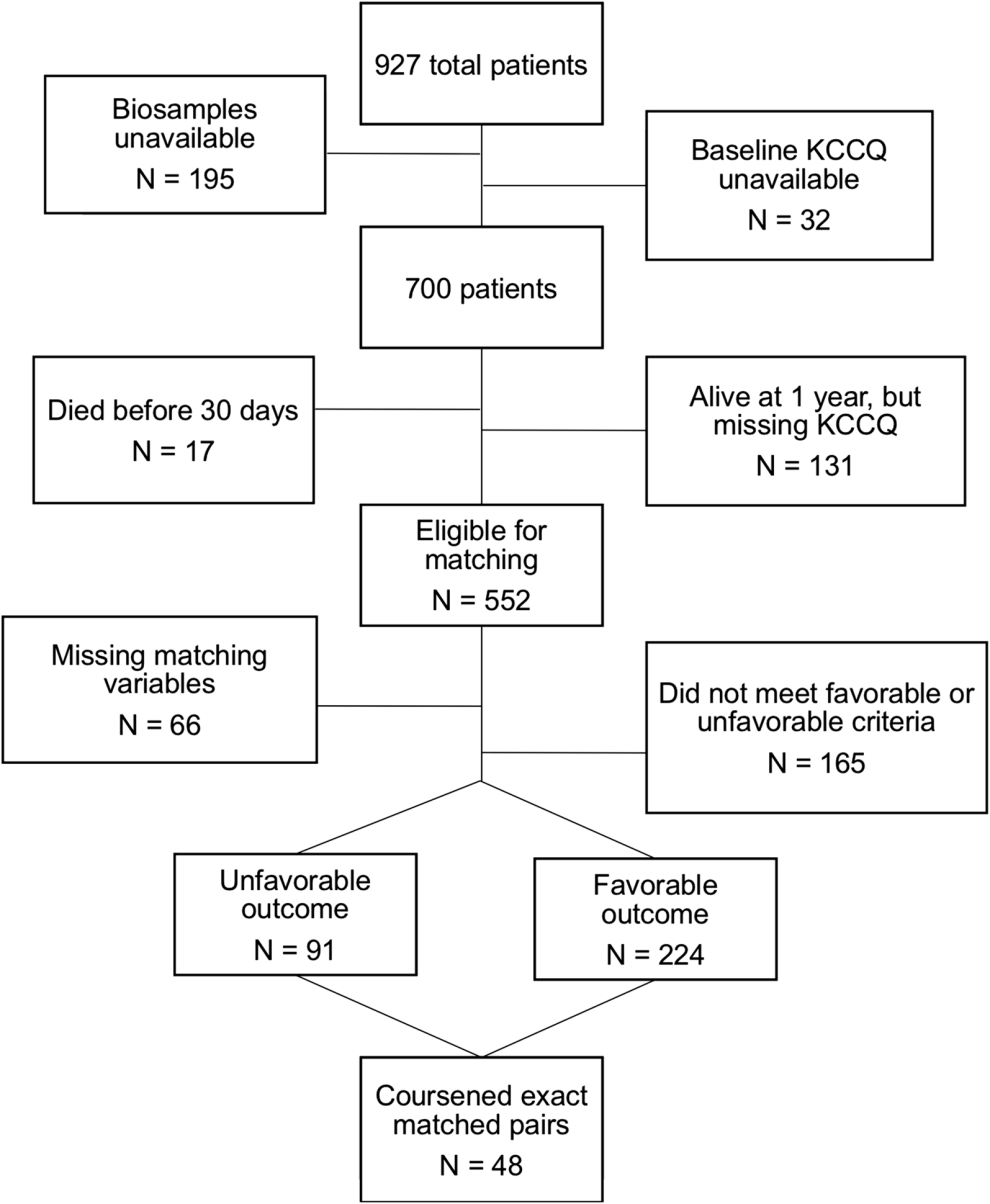
Flow chart of patients in the initial observational cohort through coarsened exact matching. The current study cohort includes the 48 pairs of patients matched from the unfavorable and favorable outcome groups

The original cohort was 927 patients. Sample selection for this retrospective case–control study was based on availability of biospecimens appropriate for RNA extraction, as well as documentation of baseline and 1-year Kansas City Cardiomyopathy Questionnaire (KCCQ) scores and 1-year survival status (Fig. 1). The KCCQ is a disease-specific health status measure that is a reliable, responsive, and valid measure of symptoms, functional status, and QoL in patients with AS [19, 20]. KCCQ scores correlate with New York Heart Association (NYHA) heart failure symptom classification scale; and changes in the KCCQ of 5, 10, and 20 points correlate with small, moderate, and large clinical change, respectively [19, 21]. Exclusion criteria were 1) death within 30 days of the procedure, to eliminate procedure-related mortality events; 2) alive at one year but lacking either baseline or 1-year KCCQ data; and 3) the absence of pre-TAVR biospecimens.

The remaining individuals were divided into three groups: Those who met a pre-defined favorable outcome, unfavorable outcome, or neither. Favorable outcome was defined as: Alive at 1 year with KCCQ score ≥ 60 AND at least moderate (≥ 10 points) improvement from pre-TAVR KCCQ score. Unfavorable outcome was defined as: Death within 1 year after TAVR or alive at 1 year but 1-year KCCQ score ≥ 10 points lower than initial KCCQ score [6, 9]. The goal was to obtain 50 matched subjects from the favorable and unfavorable groups for subsequent gene expression analyses using coarsened exact matching (CEM) [22]. As opposed to propensity risk matching, which reduces multiple risk factors to a single variable, CEM allows for matching between groups along multiple variables of interest and has been shown to achieve better balance and have lower mean square error as compared to propensity risk matching [22]. The groups were matched along “coarsened” clinical covariates including age (60–70, 71–80, 81–90, 90 +), gender (m/f), baseline KCCQ score (within 5 points), reason for TAVR (inoperable, very high risk, high risk, moderate risk), grip strength (quartiles stratified by gender), and gait speed (quartiles). Additional cardiovascular variables and other comorbidity details were not included as they are already incorporated into the reason for TAVR, which is based on the STS score that includes variables such as: extent of coronary artery disease, left ventricular function, additional valvular disease, arrhythmia, diabetes, renal disease, pulmonary disease, cerebrovascular disease, prior cardiothoracic surgery.

### Demographic, biometric, and laboratory covariates

Age, gender, race, and body mass index (BMI) were collected upon enrollment in the larger observational cohort study at each participating site. Additional data collected at this time included: medical comorbidities, immunosuppression, smoking status, and cardiovascular disease variables necessary for STS score calculation.

Individual components of frailty assessments, including gait speed, grip strength, activities of daily living (ADLs), and albumin level were collected by research staff at each study site. Gait speed was determined by the best of three timed 5-m walks. Grip strength was measured by dynamometer and scored as the best of two trials. The ability to perform ADLs was captured via Katz’s Index of Independence in Activities of Daily Living questionnaire. Individuals were classified as dependent if assistance in any of the 6 ADLs was required. Sites were instructed to record the albumin level (g/dL) obtained closest to the TAVR procedure as measured by their local laboratory.

The Columbia Frailty Score (CFS) was based on the frailty score in Green et al. on a scale of 0–12 [23]. Briefly, results of gait speed, grip strength, and albumin level were divided into quartiles and scored from 0 to 3 (best to worst). ADLs were dichotomized into independent versus dependent (i.e. assistance necessary in at least 1 or more of the ADLs) and scored as 0 or 3.

The STS score used to decide “Reason for TAVR” was calculated by each site prior to TAVR as required by the international TVT registry. To ensure consistency in STS calculation, the STS scores used in the analyses here were recalculated for each individual from raw baseline data.

### Gene expression

Venous blood whole blood was collected into PAXgene RNA tubes and frozen at −70 C before extraction (Qiagen RNeasy). Using high-efficiency mRNA-targeted library synthesis (Lexogen QuantSeq 3′ FWD), 300 ng of RNA was converted to cDNA. Libraries were then sequenced in multiplex on an Illumina HiSeq 4000 instrument (Illumina Inc., San Diego, CA) targeting > 10 million 65 bp reads per sample (achieved mean = 14 million). Reads were mapped to the reference human transcriptome sequence using the STAR aligner (average mapping rate 92.4%, 12.5 million mapped reads).

### Analysis

Gene-specific read counts were normalized to transcripts per million total mapped reads and log2-transformed for linear statistical model analyses estimating the magnitude of differential gene expression in unfavorable vs favorable outcome while adjusting for age, gender, race/ethnicity, BMI, systemic steroid use, other immune suppression, diabetes status, STS score, and CFS. The STS and CFS scores were both z-score adjusted. The covariates were selected to be consistent with previous leukocyte gene expression studies in controlling for factors that generally affect expression of the pro-inflammatory and innate antiviral system components of the CTRA profile (i.e. age, sex, race/ethnicity, smoking status). They were also selected to control for additional clinical variables that could plausibly affect inflammatory and/or antiviral gene expression in this specific context (i.e., immunosuppression, systemic steroid use, and diabetes status), as well as control for additional factors that contribute to risk for poor outcome after TAVR (STS and CFS). Robust standard errors for linear model parameters were estimated by bootstrap resampling of linear model residual vectors, which controls for correlation among genes in computing results for multi-gene composite measures. The inflammatory composite score was defined by 19 inflammatory indicator genes (*IL1A, IL1B, IL6, IL8/CXCL8, TNF, PTGS1, PTGS2, FOS, FOSB, FOSL1, FOSL2, JUN, JUNB, JUND, NFKB1, NFKB2, REL, RELA, and RELB*); Type I IFN activity was defined by a composite score of 34 indicator genes (*GBP1, IFI16, IFI27, IFI27L1-2, IFI30, IFI35, IFI44, IFI44L, IFI6, IFIH1, IFIT1-3, IFIT5, IFIT1B, IFITM1-3, IFITM4P, IFITM5, IFNB1, IRF2, IRF7-8, MX1-2, OAS1-3, OASL, JCHAIN, IGLL1, and IGLL3P*) [24–26]. Individual genes were not tested for statistical significance because this study was not designed or powered to detect gene-specific differential expression; the goal of this work was to explore canonical cellular pathways as opposed to isolated gene transcripts.

To assess the role of transcriptome control pathways, the Transcription Element Listening System (TELiS) promotor-based informatics analysis was applied to all genes demonstrating a ≥ 1.25-fold difference in average expression in unfavorable versus favorable TAVR outcome groups. Analyses were focused on immune response transcription factors involved in inflammation (Nuclear Factor κB [NF-κB], Activator Protein 1 [AP-1]) and Type I IFN response (Signal Transducer and Activator of Transcription 1 [STAT1], interferon-stimulated response element [ISRE]). TELiS analyses were conducted as previously described [27], with statistical testing derived from bootstrap standard errors controlling for covariation among genes.

## Results

### Sample characteristics

Of the original 927 cohort, 195 were missing biosample data, 32 were missing baseline KCCQ data, 17 patients died within 30 days of the procedure, and 131 patients alive at one year were missing follow up KCCQ results, leaving 552 patients available for CEM. Sixty six patients were missing variables necessary for matching. Of the remaining 486 patients, 224 had a favorable outcome, 91 had an unfavorable outcome, while 165 did not meet criteria for either defined outcome. CEM successfully matched 96 patients, with 48 patients per outcome (Fig. 1). RNA extraction resulted in poor yield in two cases, one from each outcome group. In the remaining 94 individuals, the average age was 81.3 ± 8.7, 50% were female, and the population was predominantly white. As shown in Table 1, the groups were evenly matched with respect to demographic and biometric data. The only exception was the gait speed assessment, in which a significantly higher number of individuals in the favorable outcome group were unable to walk at baseline. In the unfavorable outcome group, 29.8% were alive at 1 year.

**Table 1:** Demographics and baseline characteristics of the study cohort

	Unfavorable outcome (n = 47)	Favorable outcome (n = 47)	*P value*
Female	53.2%	46.8%	0.54
Age	82.0 ± 7.8	80.7 ± 9.6	0.49
White race	94%	100%	0.08
Diabetes	45%	30%	0.14
Systemic steroids	11%	8.5%	0.73
Other immunosuppression	6.4%	4.2%	0.65
BMI^a^	28.8 ± 8.1	28.1 ± 6.9	0.64
Smoking status			0.71
Current	2.1%	4.3%	
Former	57.4%	60.0%	
Never	38.3%	36.2%	
Reason for TAVR			0.16
Inoperable/extreme risk	12.7%	2.1%	
High risk	61.7%	72.3%	
Intermediate risk	19.1%	23.4%	
Other	6.4%	2.1%	
Gait speed			
Unable to walk	2%	17%	0.01
5 M walk speed (s)	9.4 ± 0.67	8.3 ± 4.4	0.69
Grip strength			
Men (kg)	25.6 ± 6.6	28.3 ± 10.7	0.31
Women (kg)	14.3 ± 6.4	14.7 ± 4.7	0.79
Baseline KCCQ^b^ score	45.2 ± 18.5	49.8 ± 20.6	0.54
STS^c^ score	5.4 ± 3.4	4.8 ± 3.2	0.34
Columbia Frailty score	5.0 ± 2.6	5.1 ± 3.2	0.85

Results presented as percent or mean ± SD

^a^BMI = Body mass index

^b^KCCQ = Kansas City Cardiomyopathy Questionnaire

^c^STS = Society for Thoracic Surgeons

### Pre-specified inflammatory & interferon gene composites

**Fig. 2:**
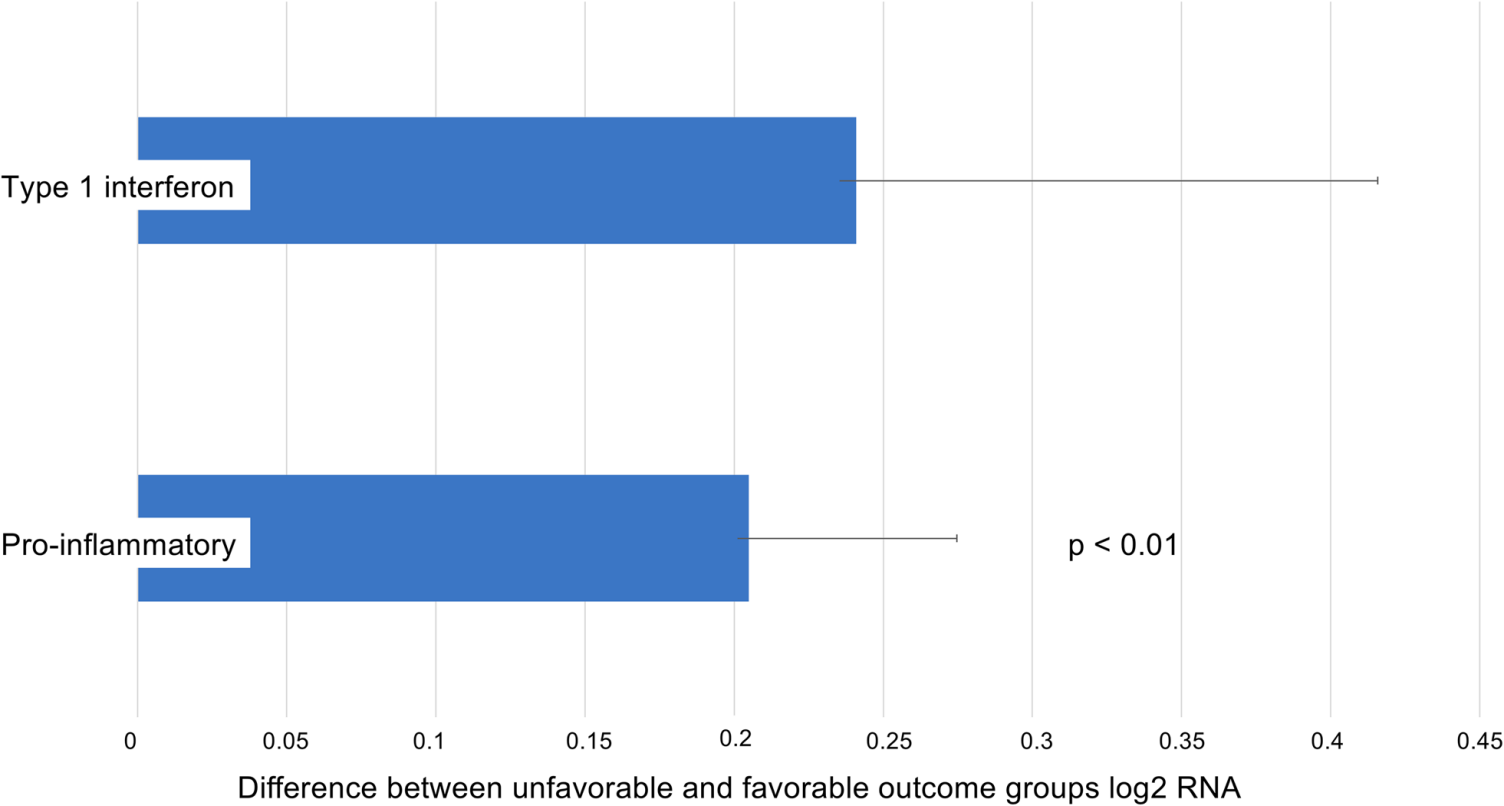
Observed inflammatory and innate antiviral system gene expression contrast between patients with favorable versus unfavorable outcomes after TAVR. The composite gene expression of the 19 pre-specified pro-inflammatory genes was significantly greater in the unfavorable as compared to the favorable outcome group (0.205 log2 RNA ± SE 0.07, p < 0.01). There was no significant difference in composite gene expression of the 34 pre-specified Type 1 interferon genes (0.241 log2 RNA ± SE 0.17, p = 0.18). TAVR = transcatheter aortic valve replacement

After controlling for the covariates, individuals with unfavorable outcomes had significantly higher average expression of the 19 pro-inflammatory indicator genes than did favorable-outcome controls (0.205 log2 RNA ± SE 0.07, p < 0.01); there was no difference across groups in the average expression of the 34 Type I IFN-related indicator genes (0.241 ± 0.17, p = 0.18) (Fig. 2). Additionally, no sex-specific nor race/ethnicity-specific differences were seen in either the pro-inflammatory or Type I IFN-related indicator genes as they relate to outcomes.

### Transcriptome control pathways

**Fig. 3:**
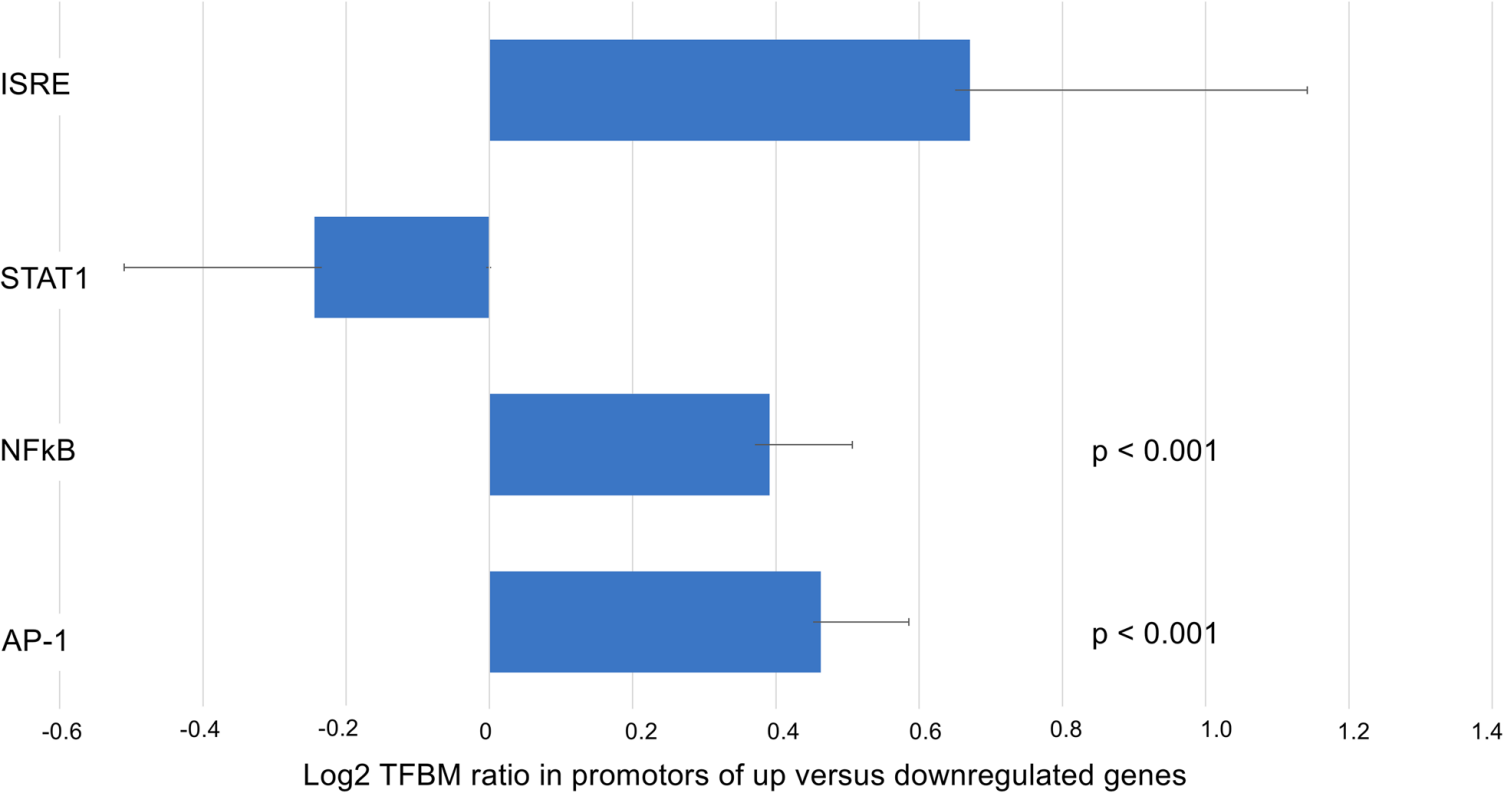
Difference in activity of inflammatory and innate antiviral system transcription control pathways in patients with favorable versus unfavorable out6comes after TAVR. TELiS promotor-based bioinformatics analyses were utilized for all genes showing > 25% differential expression between favorable and unfavorable outcome groups. In 6,036 gene transcripts (5257 up-regulated and 778 down-regulated), results indicated increased activity of pre-specified inflammation-related transcription factors NF-κB (mean log ratio 0.39 ± 0.12, p < 0.001 log2 transcription factor binding motif ratio in promotors of up vs. downregulated genes) and AP-1 (mean log ratio 0.42 ± 0.12, p < 0.001) but no significant difference in pre-specified Type I interferon-related transcription factors ISRE (mean log ratio 0.67 ± 0.47, p = NS) or STAT1 (mean log ratio -0.24 ± 0.27, p = NS). AP-1 = Activator Protein 1; IFN = interferon; ISRE = Interferon-stimulated Response Element; NF-κB = Nuclear Factor-κB; TAVR = transcatheter aortic valve replacement; TELiS = Transcription Element Listening System; TFBM = Transcription factor binding motif; STAT1 = Signal Transducer and Activator of Transcription 1

In promoter-based bioinformatics analyses of the 6,036 gene transcripts with ≥ 25% difference in average expression across groups (5257 up-regulated and 778 down-regulated), results indicated increased activity of pre-specified inflammation-related transcription factors NF-κB (mean log ratio 0.39 ± 0.12, p < 0.001 log2 transcription factor binding motif ratio in promotors of up vs. downregulated genes) and AP-1 (mean log ratio 0.42 ± 0.12, p < 0.001) but no significant difference in pre-specified Type I IFN-related transcription factors ISRE (mean log ratio 0.67 ± 0.47, p = NS) or STAT1 (mean log ratio -0.24 ± 0.27, p = NS) (Fig. 3).

## Discussion

In this case–control study exploring the relationships of pro-inflammatory and Type I IFN gene expression as it relates to long-term outcomes of older adults after TAVR, we found that upregulation of pro-inflammatory gene expression at baseline was independently associated with the composite outcome of mortality or diminished quality of life one year after the procedure. This finding was supported by both increased expression of a pre-specified composite of 19 pro-inflammatory gene transcripts and by bioinformatic analyses of inflammation-associated transcription factors, NF-κB, and AP-1, in genes showing empirical differences in expression in individuals with unfavorable outcomes. Although decreased Type I IFN response gene expression is an expected pattern associated with aging, we did not find this pattern to be significantly related to adverse long-term outcomes in older adults undergoing TAVR. Overall, these findings suggest that increased activity of pro-inflammatory genes may be an important predictor of outcomes as well as a possible therapeutic target for intervention.

Age-associated inflammation has been implicated in numerous chronic diseases. Driven by activity of the transcription factor NF-κB and AP-1, inflammatory cytokine dysregulation is seen in cardiovascular diseases such as aortic stenosis and atherosclerotic coronary artery disease, as well as aging-associated conditions like frailty [28]. Therefore, in older adults presenting for TAVR, it is unsurprising that baseline serum markers of inflammation are higher than in age matched controls [29]. Prior work has shown that elevated non-specific inflammatory markers prior to TAVR, including CRP and IL-6, have been associated with increased 1-year mortality after TAVR [30]. Although the gene expression of inflammatory pathways has not been investigated with respect to outcomes after TAVR, dysregulation of these systems has been implicated in poor resilience to medical stressors such as injury, chemotherapy, and surgery [31, 32]. Studies suggest that enhanced inflammatory gene expression may be related to poor outcomes after kidney transplant in older adults [31], and impaired fracture healing [33], while enhanced inflammatory gene expression predicts more fatigue after breast cancer surgery and chemotherapy [34, 35]. Here, we show that gene expression related to common inflammatory gene expression pathways is related to adverse long-term quality of life and mortality outcomes after TAVR.

One hallmark of aging is altered immune system activity. In addition to increased inflammation, age-associated changes in innate immune activity impair the system’s ability to mount efficient responses to new pathogens and diminish responses to vaccines [36]. The subsequent decline in immune-system responsivity has been implicated in increased susceptibility to infection and malignancy [37], as well as all-cause mortality [38]. With respect to TAVR outcomes, decreased baseline T2 helper cell count, a nonspecific marker of immune activity, has been associated with mortality after TAVR [30]. Diminished gene expression of antiviral and antibody mediated responses has also been implicated in poor outcomes after kidney transplant in older adults [31] and decreased leukemia-free survival in hematopoetic stem cell transplant recipients [32]. In contrast, we did not find any significant relationship between TAVR outcome and expression of antiviral and antibody-related genes in circulating blood cells. Innate antiviral gene regulation is controlled by distinct transcription factor systems from inflammation [39] and the present data suggest that TVAR outcomes are selectively associated with the pro-inflammatory sytem, whereas the innate antiviral system showed no association with TAVR outcomes. This may indicate that viral replication is not a significant determinatnt of TAVR outcomes whereas alterations in tissue homeostasis associated with inflammation play a more substantial role in successful recovery. It is also possible that previous associations of antiviral gene regulation with kidney and hematopoietic stem cell transplants may reflect the greater levels of disease- or treatment-related immunosuppression observed in those conditions compared to patients with severe aortic stenosis undergoing TAVR.

Both immune senescence and chronic inflammation are thought to be part of the pathophysiologic processes underlying the increased vulnerability seen in frail individuals [40]. This is one of the first studies to explore different elements of the immune system and how they relate to outcomes after a cardiovascular procedure. Our data shows that increased pro-inflammatory but not anitiviral gene expression at baseline prior to TAVR is associated with adverse outcomes in older adults undergoing TAVR. Surprisingly, we found this association to be independent of frailty status. Although additional work to explore the relationship between inflammation and frailty in a cardiovascular population is necessary, this suggests that there may be benefit to intervening upon pro-inflammatory states to improve outcomes regardless of frailty status.

Pro-inflammatory gene expression may be a modifiable element of baseline procedural status. Anti-inflammatory therapies like optimizing sleep [41], increasing physical activity [42], participating in mindfulness programs [43, 44], inflammation-reducing medication administration [45], and allogenic mesenchymal stem cell administration [46] have all shown potential to improve inflammatory gene expression profiles. Specifically, between six to twelve weeks of participation in mind–body therapies, such as tai chi, yoga, and meditation favorably modify pro-inflammatory leukocyte gene transcription pathways [47]. Six weeks of cognitive behavioral therapy (CBT) has a similarly beneficial impact on inflammatory and immune system gene expression [43] and is associated with favorable QoL outcomes [48]. Finally, short term administration of pharmacological therapy to reduce inflammation or sympathetic system activity (e.g. propranolol) improved inflammatory and innate immune system gene expression and was associated with better outcomes after hematopoietic stem cell transplant [49]. Furthermore, in light of the upregulation of NF-kB and AP-1 in TAVR patients with unfavorable outcomes, modulating the activity of these transcription factors may provide an additional therapeutic target for pre-procedural interventions [16]. Therefore, using inflammatory gene expression as a modifiable biomarker of risk prior to TAVR may provide a specific and measurable target for peri-procedural interventions designed to improve long term outcomes.

The findings of this study should be interpreted in the context of its limitations. First, we did not compare baseline gene expression in a TAVR population with age-matched control subjects. It therefore remains unknown how inflammatory and immune gene expression profiles in the severe AS population differ from community dwelling older adults. Nevertheless, in the TAVR population, upregulated inflammatory gene expression was associated with worse outcomes after the procedure. Because of the sample size and infrequency of peri-procedural deaths, we also excluded individuals with peri-procedural mortality within 30 days. The role of inflammatory and immune gene expression in peri-procedural mortality events thus remains unexplored. Additionally, although likely related to pre-procedural inflammatory and antiviral gene expression, we did not explore peri-procedural complications such as infection, as our goal was to assess long-term outcomes of importance to older adults, namely survival with good quality of life. Finally, as a case–control study, we sampled outcome extremes and were unable to capture the relationship of gene expression profiles to a broader range of outcomes. However, only 18% of the sample did not fall into the favorable or unfavorable categories we defined.

In conclusion, long-term outcomes after TAVR are thought to be driven by baseline medical comorbidities and complexities of aging as opposed to consequences of the procedure itself. In addition to recognizing factors that increase procedural vulnerability (e.g., elevated STS score, poor vascular access, higher risk of bleeding), identifying baseline characteristics that increase vulnerability to adverse long-term outcomes will help guide discussions regarding anticipated procedural results and facilitate more specific risk–benefit calculations. In this study, we identified pro-inflammatory gene expression as a marker of increased risk for adverse long-term TAVR outcomes. Future studies will explore whether targeted peri-procedural therapies designed to modulate pro-inflammatory gene expression may improve resilience and promote more favorable long-term procedure outcomes of importance to older adults.

## Data Availability

The datasets used and/or analysed during the current study are available from the corresponding author on reasonable request.

## Abbreviations

ADL: Activities of daily living
AP-1: Activator Protein 1
AS: Aortic stenosis
BMI: Body mass index
CEM: Coursened exact matching
CFS: Columbia Frailty Score
CVD: Cardiovascular disease
hsCRP: High sensitivity C-reactive protein
IFN: Interferon
IL: Interleukin
ISRE: Interferon-stimulated Response Element
KCCQ: Kansas City Cardiomyopathy Questionnaire
NF-κB: Nuclear Factor-κB
NYHA: New York Heart Association
QoL: Quality of life
SASP: Senescence-associated secretory phenotype
STAT1: Signal Transducer and Activator of Transcription 1
STS: Society for Thoracic Surgeons
TAVR: Transcatheter aortic valve replacement
TELiS: Transcription Element Listening System
